# Deep learning for de-convolution of Smad2 versus Smad3 binding sites

**DOI:** 10.1186/s12864-022-08565-x

**Published:** 2022-07-20

**Authors:** Jeremy W.K. Ng, Esther H.Q. Ong, Lisa Tucker-Kellogg, Greg Tucker-Kellogg

**Affiliations:** 1 Department of Biological Sciences, National University of Singapore, Singapore, Singapore; 2 Cancer and Stem Cell Biology, and Centre for Computational Biology, Duke-NUS Medical School, Singapore, Singapore; 3 Computational Biology Programme, Faculty of Science, National University of Singapore, Singapore, Singapore

**Keywords:** Machine learning, Transcription regulation, Feature engineering

## Abstract

**Background:**

The transforming growth factor beta-1 (TGF *β*-1) cytokine exerts both pro-tumor and anti-tumor effects in carcinogenesis. An increasing body of literature suggests that TGF *β*-1 signaling outcome is partially dependent on the regulatory targets of downstream receptor-regulated Smad (R-Smad) proteins Smad2 and Smad3. However, the lack of Smad-specific antibodies for ChIP-seq hinders convenient identification of Smad-specific binding sites.

**Results:**

In this study, we use localization and affinity purification (LAP) tags to identify Smad-specific binding sites in a cancer cell line. Using ChIP-seq data obtained from LAP-tagged Smad proteins, we develop a convolutional neural network with long-short term memory (CNN-LSTM) as a deep learning approach to classify a pool of Smad-bound sites as being Smad2- or Smad3-bound. Our data showed that this approach is able to accurately classify Smad2- versus Smad3-bound sites. We use our model to dissect the role of each R-Smad in the progression of breast cancer using a previously published dataset.

**Conclusions:**

Our results suggests that deep learning approaches can be used to dissect binding site specificity of closely related transcription factors.

**Supplementary Information:**

The online version contains supplementary material available at (10.1186/s12864-022-08565-x).

## Introduction

Transforming growth factor-beta (TGF *β*) signaling contributes to a wide range of cellular behaviors in both normal and tumor settings. TGF *β* plays essential roles in differentiation [1, 2], epithelial-mesenchymal transition (EMT) [3, 4], cytostasis [5], cell migration [6], angiogenesis [7] and wound healing [8]. Its role in carcinogenesis has been described as paradoxical because TGF *β* can act as either a tumor suppressor or a driver of cancer progression depending on context [9, 10]. The paradoxical role of TGF *β* in cancer biology has led to a growing body of data documenting molecular co-factors that determine the different TGF *β* outcomes. However, an unmet need remains to re-analyze prior TGF *β*-pathway data according to what is now known about specific molecular determinants.

The canonical pathway of TGF *β*-1 signaling is initiated when an extracellular TGF *β*-1 ligand binds and induces dimerization of the TGF *β* receptor, which then phosphorylates one of the R-Smad proteins Smad2 or Smad3. The phosphorylated R-Smad forms a complex with the common partner (co-Smad) Smad4 and translocates to the nucleus to regulate the expression of target genes [11, 12]. Activation of R-Smads is partly regulated by dynamic phosphorylation-dependent shuttling of R-Smad complexes between the cytoplasm and the nucleus [12, 13].

Although both Smad2 and Smad3 can be phosphorylated by the same receptor, activation of different R-Smads often leads to different regulatory outcomes. For example, in the metastatic breast cancer cell model MDA-MB-231, Smad2 knock-down led to a more aggressive phenotype, while Smad3 knock-down led to a lag in tumor initiation, suggesting that Smad2 and Smad3 have opposing effects on disease progression [14]. Another study in HaCaT cells showed that Smad3 was responsible for driving cell-cycle arrest [15]. These Smad2- and Smad3-specific signaling outcomes have been further traced to Smad-specific binding of transcription factors to the R-Smad complex [16]. Smad binding partners affect which transcription sites are bound by the R-Smad complex because the R-Smads by themselves have low DNA binding affinity (1.1 × 10 ^−7^M) by electroshift mobility assay [17]).

Since Smad-driven genome regulation is mediated through chromatin binding, it should be possible to distinguish Smad2- from Smad3-driven regulation using genome-wide binding measurements of Smad binding elements (SBEs). However, direct genome-wide measurement of specific R-Smad binding is limited by the lack of Smad2-specific antibodies for ChIP-Seq or similar experiments. This is a challenge that pervades the Smad signaling literature (most studies simply refer to “Smad2/3” signaling), but is particularly challenging for genome-binding measurements. Consequently, most ChIP-seq studies of Smads use a high quality pan-Smad2/3 antibody and are unable to distinguish the regulation by the different Smads. Efforts to measure Smad-specific genomic binding directly, such as by transfection of Smad fusion proteins, or CRISPR knock-out of either Smad2 or Smad3, would perturb R-Smad abundance and disrupt the nucleo-cytoplasmic feedback dynamics [13].

An experimental solution to this challenge would be to provide cells with epitope-tagged Smads in a native cis-regulatory environment. This can be accomplished using methods such as the BAC TransgenOmics platform [18], in which epitope-tagged BAC transgenes are introduced into mammalian cells, preserving proximal cis-regulatory elements. More recent genome editing approaches, such as CRISPR/Cas9, can also be used for epitope tagging in the genome itself [19]. Such an experimental approach, however, would not disambiguate Smad binding in previously generated data. The limited information available about Smad2-specific and Smad3-specific effects would be more useful if it could help provide Smad-specific attribution for the vast amounts of non-specific information already collected regarding Smad2/3 combined effects.

Recent advances in machine learning have enabled the use of models trained on existing data to perform transcription factor binding site (TFBS) prediction. The power of such models was demonstrated in the ENCODE-DREAM challenge, where teams competed to develop models for cell type-specific TFBS prediction using ATAC-seq data. The top entries such as Anchor [20], Catchitt [21], and FactorNet [22] were able to accurately predict the binding sites of transcription factors in cell types not included during training. Despite the promise of cell type-specific TFBS prediction using machine learning, model performance varies widely, partly due to differences in the quality of training data available. More recently, neural networks such as Deepbind[23] and DeepTF [24] are being used to perform TFBS prediction. While Convolutional Neural Networks (CNNs) were initially developed for use on image data, CNNs have also been used for feature selection on non-image data, as exemplified by methods such as DeepInsight [25] and DeepFeature [26]. However, most machine learning approaches to TFBS prediction have been evaluated on widely studied transcription factors such as REST and CTCF, where large amounts of data are available for model training. To the best of our knowledge, no model has been developed to disambiguate R-Smad binding sites.

In this study, we combine experimental genome-wide measurement of Smad-specific binding sites with deep learning to disambiguate genome-wide Smad2 and Smad3 binding in new and existing data. In order to experimentally distinguish Smad2 and Smad3 target sites, Smad2 and Smad3 fusion proteins were transfected into the breast cancer cell line MDA-MB-231 in a native cis-regulatory environment as BAC transgenes [18]. ChIP-seq was then performed using the fusion tags to identify binding regions of each R-Smad. Geometric analysis of the binding regions identified sequence-dependent structural features, suggesting that sequence-based learning could distinguish R-Smad-specific binding. Using the collected sequences as training data, we developed a deep learning model to classify Smad2- and Smad3-binding regions. We applied this model to the problem of attributing Smad2- versus Smad3-binding for regions of known pan-Smad2/3 antibody binding. Specifically, we re-analyzed a public ChIP-Seq data set that had been generated using a pan-Smad2/3 antibody, and our method inferred potential Smad2- and Smad3- driven genomic regulation. This study represents a proof of concept for the broader use of deep learning to resolve the specificity of genomic regulation driven by closely related transcription factors.

## Results and discussion

### LAP-tagged r-Smad BAC system is able to recapitulate native TGF *β* signaling

Immunoblots confirmed the presence of the LAP-Smad, which resolved at a higher molecular weight due to the presence of the LAP tag. The LAP-Smad was detected together with the endogenous Smad of interest when cell lysate was immunoblotted against a specific Smad; LAP-Smad2 at 85kDa could be detected together with the endogenous Smad2 at 58kDa when immunoblotted with Smad2 antibody. The LAP-Smad2 was also detected at the same 85kDa size when immunoblotted with GFP antibody. No LAP-Smad3 was detected in the MDA-Smad2 cell lysate, and vice versa, indicating that there was no cross interaction (Additional file 1).

**Fig. 1:**
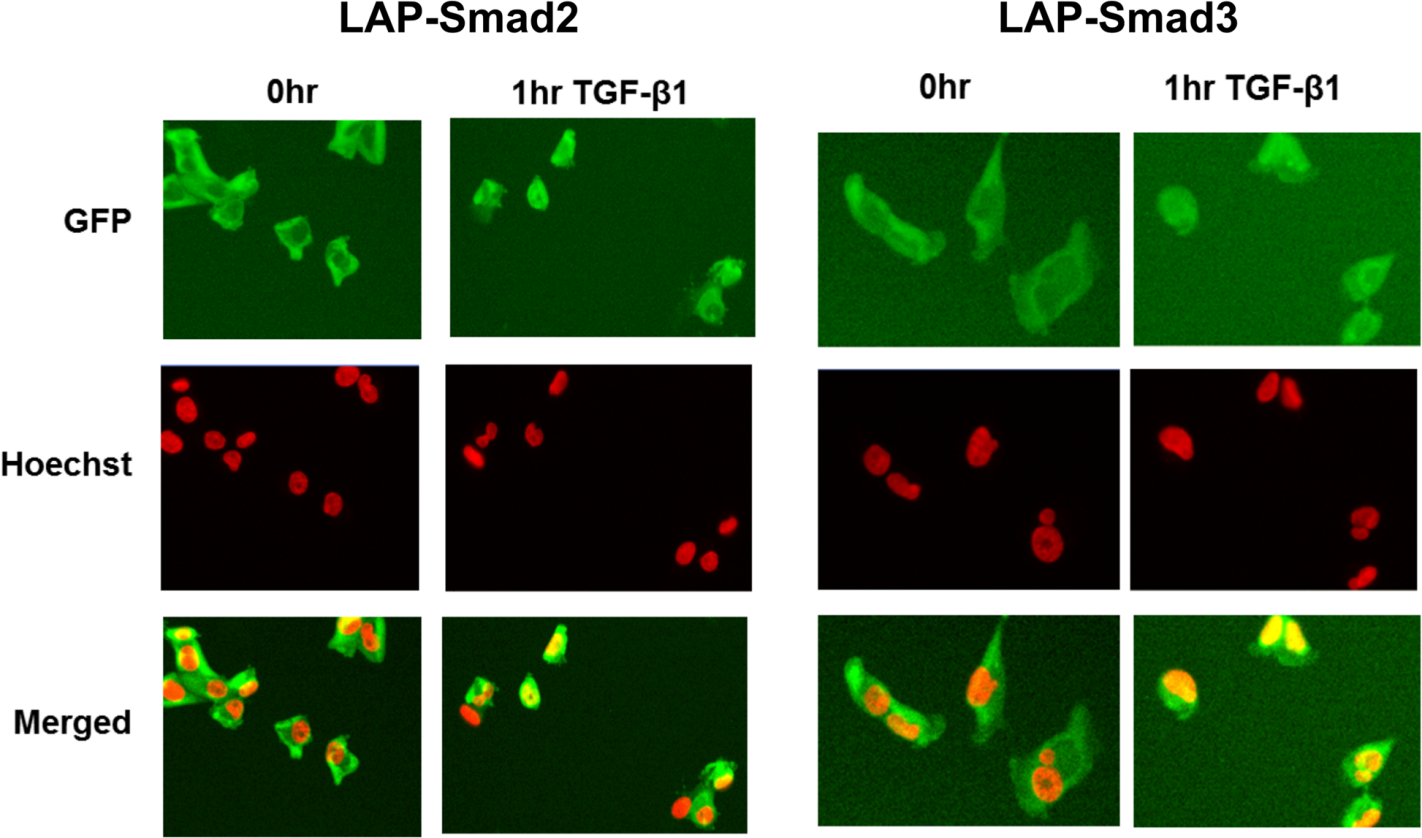
Translocation of LAP-Smads from the cytoplasm to the nucleus upon stimulation with TGF *β*-1, shown via high content imaging in MDA-MB-231 cells. Left, LAP-Smad2, right LAP-Smad3. Cell nuclei were stained with red Hoechst stain (DAPI channel). Green GFP stain (FITC channel) showed predominantly cytoplasmic localization of LAP-Smads in the absence of TGF *β*-1 stimulation, and translocation to the nucleus after TGF *β*-1 stimulation

To illustrate the functionality of the LAP-Smad, high content analysis imaging was performed with anti-GFP antibody to demonstrate the translocation of LAP-Smad2 and LAP-Smad3 upon TGF *β*-1 stimulation. In the absence of TGF *β*-1, the LAP-Smad2 and LAP-Smad3 were mainly localized in the cytoplasm. Translocation of LAP-Smad into the nucleus was observed 1 hour after 10ng/mL TGF *β*-1 stimulation (Fig. 1).

### LAP-tagged r-Smad BAC ChIP-seq shows good concordance with native ChIP-seq

We used an approach similar to the Irreproducible Discovery Rate [27] of ENCODE for comparing the peaks called using LAP-tagged Smad3 and native Smad3 ChIP-seq generated in-house. Briefly, peaks were called using MACS2 using the default parameters and a cut-off q-value of 0.05 in both experiments. The distance from each peak obtained from the Smad3 ChIP of MDA-MB-231 to the nearest peak found with the GFP-ChIP of MDA-Smad3 was calculated using GenomicRanges [28]. Finally, the distance to the nearest peak was visualized as a function of the *p*-value of the peak. If the *p*-value indicates confidence, then we would expect peaks with higher *p*-value to have shorter distances between peaks (i.e., greater overlap between both ChIP experiments). Indeed, we found that although LAP-tagged Smad3 allowed a greater number of peaks to be called, there was still good concordance between peaks called in native Smad3 as well as LAP-tagged Smad3 (Additional file 2). In particular, we observed that peaks with *p*-values of less than 10 ^−20^ in our MDA-Smad3 ChIP overlapped a peak identified in our native Smad3 ChIP. This result suggests good concordance between a LAP-tag Smad3 ChIP-seq and native Smad3 ChIP-seq. Having established the concordance of our LAP-tag Smad ChIP, we turned to characterizing Smad2 and Smad3 bound sites.

### Characterising Smad2 and Smad3 binding sites

Earlier studies had highlighted a role of 3D conformation in determining the binding affinity of transcriptional co-regulators [29–31]. Furthermore, a recent structural study of FOXH1-driven TGF *β* signaling identified DNA shape characteristics that distinguished Smad2- versus Smad3binding complexes [32]. We took advantage of these findings to characterize key shape properties of the respective R-Smad binding sites using the R package DNAshape [33]. The binding regions of Smad2 and Smad3 obtained from our LAP-Smad ChIP were subjected to computational prediction of structural and geometric features, such as minor grove width and electrostatic potential.

**Fig. 2:**
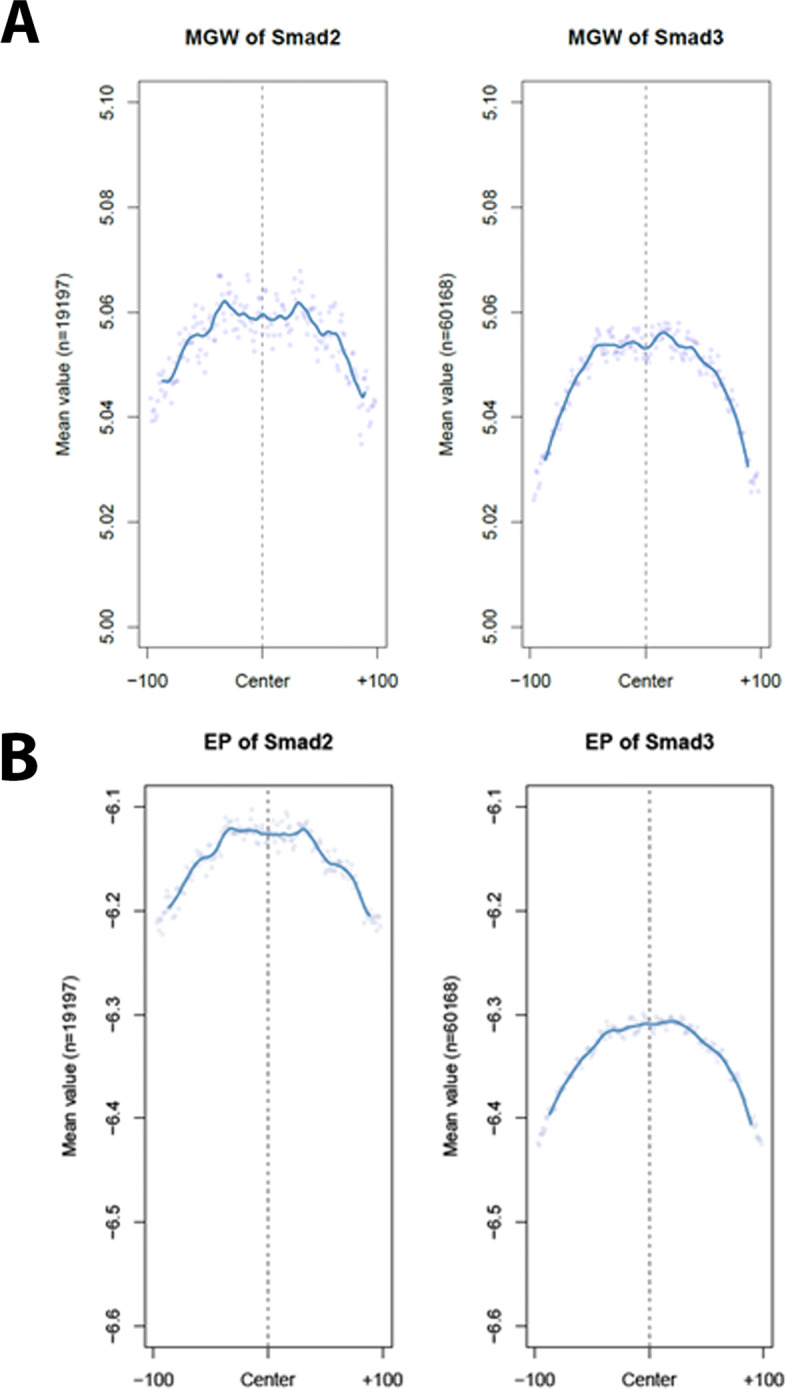
Characterization of Smad2 and Smad3 binding sites using *DNAShapeR*. **A**. Minor grove width (MGW) of Smad2-bound sites (left) and Smad3-bound sites (right). While both Smad2 and Smad3 had similar MGW at the centers of the peaks, there was a marked difference in the MGW 100 base pairs upstream and downstream of the peak center, with Smad3-bound peaks narrower than Smad2-bound. **B**. Electrostatic potential (EP) of Smad2- and Smad3-bound sites. Smad2-bound sites (left) were observed to have higher electrostatic potential when compared to Smad3-bound sites across the full 200 base pairs of each binding site

While the minor grove width (MGW) of Smad2- and Smad3-bound sites were similar at the middle of the binding peaks, we observed that the MGWs at the farthest ends (+/- 100 base pairs) of Smad3-bound peaks were narrower than for the Smad2-bound peaks (Fig. 2A). We also observed larger electrostatic potential in Smad2-bound regions as compared to Smad3-bound regions (Fig. 2B). These differences can be attributed to differences in the underlying DNA structure. The intrinsic flexiblity of DNA can be characterized along dinucleotide steps [34]: flexible steps allow for more exploration of conformational space while stiffer steps allow for less. Likewise, C:G base pairs have a larger electrostatic potential due to the presence of a partial positive charge on the amine group of cytosine. The more negative electrostatic potential observed in the narrower Smad3-bound sites is also consistent with earlier Poisson-Boltzmann calculations that show lower electrostatic potentials in structures with narrower MGW [29]. Both intrinsic flexibility and electrostatic potential contribute to sequence-dependent groove width differences [35]. Consistent with our expectation, Smad2-bound regions had an average GC content of 50.3% as compared to Smad3-bound regions with an average of 50.0% (p < 0.05, using t-test).

Biologically, the differences between Smad2- and Smad3-bound sites can be traced to the differences in transcriptional co-regulators that interact with each respective R-Smad. Motif enrichment analysis was performed to identify potential co-regulators of Smad2 and Smad3 binding. While both Smad2- and Smad3-bound promoters were enriched for MEF, Smad2-bound promoters were exclusively enriched for various basic helix-loop-helix (bHLH) transcription factors such as E2A, Tcf12, and Ascl. This is juxtaposed to the exclusive enrichment of Smad3-bound promoters for various nuclear receptors (NR). The bHLH family of recognize the E-box motif [36] comprised of the canonical CG-rich sequence CANNTG [37]. On the other hand, the NR family of transcription factors recognize the P-box motif, which comprises either AGAACA or AGGTCA [38].

Taken together, our characterization of the shape features of Smad2- and Smad3-bound sites suggests DNA sequence could potentially encode information about R-Smad specificity. Hence, we sought to build a model that enables de-convolution of Smad2 and Smad3 binding using DNA sequence. Using such a model, we seek to classify a peak identified using a pan-Smad2/3 antibody as being Smad2-bound or Smad3-bound.

### CNN-LSTM hybrid model that can distinguish between Smad2 and Smad3 binding sites

**Fig. 3:**
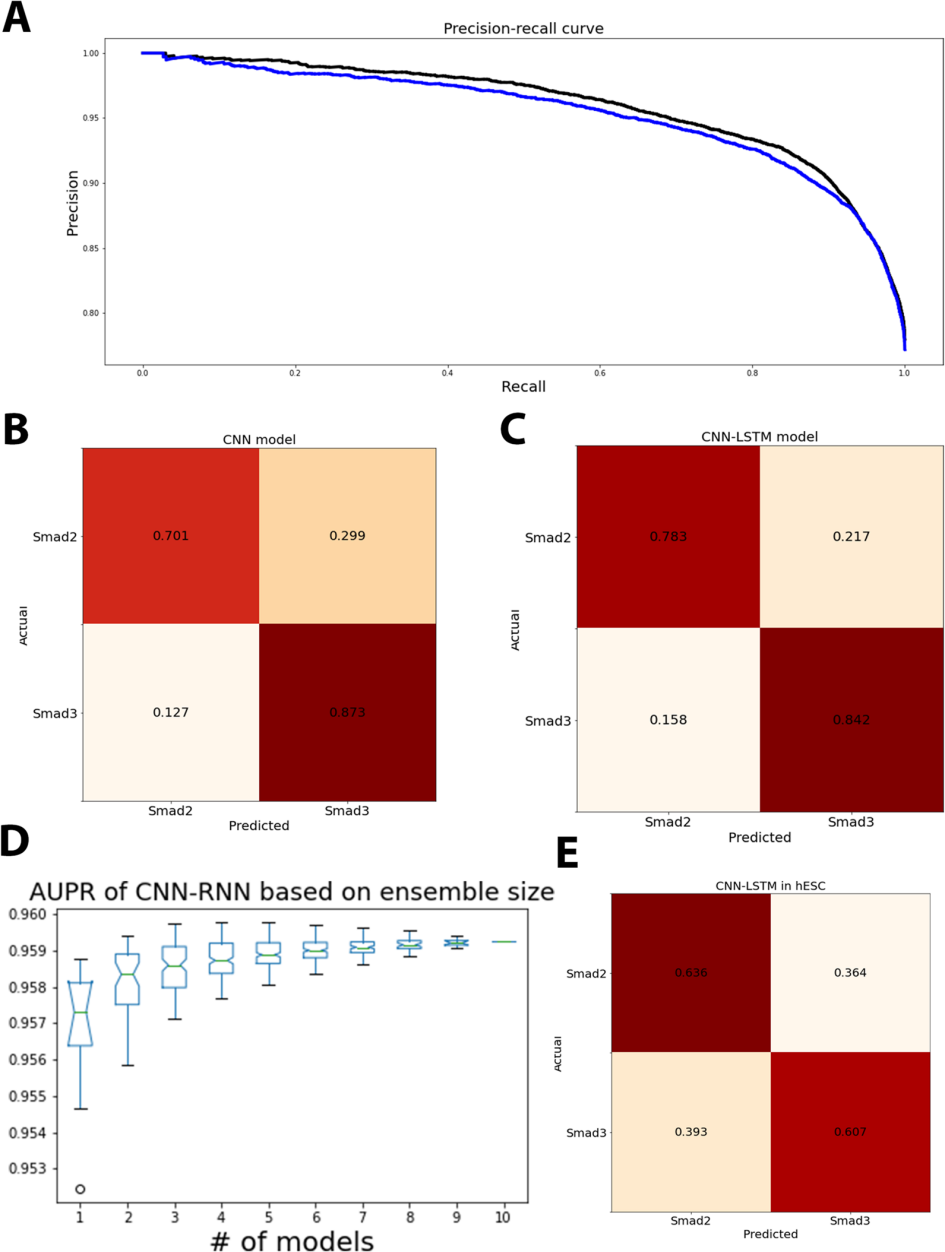
Neural networks can classify Smad-bound sites as being Smad2- or Smad3-bound.**A**. Precision recall curve of CNN (blue) and CNN-LSTM (black) models, taking the average of 10 models for final classification. An average precision of 0.95 was observed for the CNN model, as compared to the slightly higher average precision of 0.96 of the CNN-LSTM model. The model was better able to classify Smad3 (0.87) as compared to Smad2 (0.7) **B**. Confusion matrix of CNN model in classifying Smad2 and Smad3 sites. The model was able to better classify Smad3 (0.7 vs 0.87). **C** Confusion matrix of CNN-LSTM. Similar to the CNN model, the CNN-LSTM model was also better at classifying Smad3 (0.84) as compared to Smad2 (0.78), but performed better than the CNN model (as shown in A). **D**. The effect of ensemble learning on model performance evaluated using AUCPR. We evaluated the performance of increasing the number of models used from one to ten, with increase in AUCPR observed as the number of models increased. The standard deviation, indicative of stability, also decreased as more models were included in the final ensemble. **E**. Confusion matrix of Smad2/3 binding in hESC, showing model performance in a novel cell type was not included in the training dataset

Both CNNs and RNNs have been used extensively in a TFBS prediction tasks, with both yielding competitive results in various TFBS prediction tasks. We first sought to assess the suitability of each network architecture for de-convolving Smad binding sites. As shown in Fig. 3, the AUPR obtained on the testing set for both CNN and CNN-LSTM models were comparable (0.95 and 0.96, respectively) when we used 10 models for prediction. Notably, the CNN-LSTM model was able to classify Smad2-bound sites better despite the imbalanced training data, increasing the accuracy from 0.7 to 0.78 at a cost of a 0.03 decrease in the accuracy of Smad3 predictions. The improved performance of the CNN-LSTM hybrid is consistent with the finding by Lanchantin et al. [39] that a medium-sized CNN-RNN hybrid model yielded a higher AUC compared to a small CNN comprising 2 convolutional layers while having smaller standard deviations between different models. While no neural network has been previously developed for the task of de-convoluting the binding sites of closely related transcription factors, the AUPR obtained in our study is comparable with state-of-the-art TFBS methods such as Catchitt, which reported an AUPR of > 0.8 in classification of CTCF using a large training data-set [21].

We also evaluated the impact of using different numbers of models for ensemble learning. The final output probability for classification is calculated by taking the average probability from all the models used in an ensemble. This was done by enumerating all possible combinations of *N* models (where *N* is the number of models to be used for ensemble learning). As expected, increasing the number of models led to increased AUPR (Fig. 3C). Increasing the number of models also decreased the standard deviation, suggesting greater consistency in predictions between models. These findings are consistent with earlier work in machine learning that demonstrated the superiority of ensemble methods in classification tasks [21, 40]. Taken together, our results show that snapshot ensemble learning, combined with a cosine annealing training schedule, was a computationally efficient approach for increasing the performance of NN-based TFBS prediction.

To test if our model could be generalized, we tested our model on the human embryonic stem cell (hESC) dataset deposited by Kim et al. [41]. In this dataset, Kim and colleagues sought to identify Smad2 and Smad3 binding sites during embryonic development. Due to the lack of Smad2 specific antibodies, Smad2 binding sites were inferred by performing ’peak subtraction’. In brief, a pan-Smad2/3 antibody was first used to obtain a list of all Smad2/3 binding sites. A second ChIP was then performed using a commercially available Smad3-specific antibody. Finally, the Smad2 sites were identified by removing binding sites that were common in both ChIP experiments. We compared the predicted classification of Smad binding sites with the classification based on peak substraction. The results are shown in Fig. 3D. Despite the low AUPR (0.44), the confusion matrix showed that our model was able to classify Smad2- and Smad3-bound sites correctly about 60% of the time - a decrease in performance when compared to the testing dataset. However, the decrease in accuracy can be attributed to the lack of cell-specific training data, as our model was trained using Smad binding sites in a breast cell line. The dependence of model performance on the size of the training dataset has been also observed in other state-of-the-art TFBS prediction models.

### De-convolving the roles of Smad2 and Smad3 in mCF10A-MII cells

Having shown that our model is able to classify Smad-bound sites as either Smad2- or Smad3-bound with reasonable accuracy, we sought to leverage our model to investigate the relative contributions of each R-Smad in breast cancer progression. Sunquivst and colleagues performed ChIP-seq against Smad2/3 in MCF10A-MII cells to identify early and late TGF *β* (16 hours) response genes, and demonstrated a shift in Smad2/3 binding sites following sustained TGF *β* treatment [42]. However, the authors were not able to differentiate between Smad2- and Smad3-bound genes. To de-convolute the contribution of each R-Smad to breast cancer progression, we used our model to classify Smad-bound sites as either being Smad2- or Smad3-bound. This might shed light on the contributions of each R-Smad in sculpting the response of MCF10A-MII cells to TGF *β*-1. Following classification, we performed GO-enrichment to functionally characterize the Smad2- and Smad3-bound peaks in both early and late TGF *β* response.

In the early TGF *β* response, we observed an enrichment of TGF *β* signaling related pathways among Smad3 peaks (Table 1). This suggests that Smad3, and not Smad2, upregulates canonical TGF *β* target genes. This observation is corroborated by experimental evidence from the literature demonstrating the direct role of Smad3 in regulating the expression of canonical early response genes such as *Id1* and *Smad7*. For instance, Liang and colleagues demonstrated that Smad3, and not Smad2, leads to the induction of Id1 expression one-hour post treatment in MCF10A cells [43]. Likewise, Smad3 also directs the expression of Smad7 via direct binding to the promoter [44].

**Table 1:** Functional annotation of Smad2 and Smad3 bound genes in MCF10A-MII cells. RTK: receptor tyrosine kinase. ECM: extracellular matrix. NI: Non-integrin

Early		Late	
Smad2	Smad3	Smad2	Smad3
RTK signaling	RTK signaling	Neuronal System	RTK signaling
ECM reorganization		ECM reorganization	
Extra-nuclear estrogen signaling	Signaling by TGF *β* members	cell-cell communication	ECM degradation
Signaling by MET	Signaling by TGF *β* Receptor Complex	Cell junction organization	cell-cell communication
NI membrane-ECM interactions	VEGFA-VEGFR2 Pathway	Netrin-1 signaling	NI membrane-ECM interactions

Turning to the pathways regulated by each Smad following 16 hours of treatment, we observed that Smad3 targets were associated with processes involved in the re-organization of the extracellular matrix (ECM), including ECM degradation. The degradation of ECM is a crucial step during cell invasion process. On the other hand, we observed terms associated with neural development in Smad2-bound loci. A role of Smad2 in neural development has been observed in mouse models, with the Smad2 *δ* exon-3 isoform being enriched in the nuclear fraction during brain cell differentiation [45]. The process of neural development includes EMT and directed migration, and has striking resemblance to cell migration in carcinogenesis [46].

## Conclusion

In this study, we first validated a LAP-tagged R-Smad system that enables identification of Smad2- and Smad3-specific binding sites in a breast cancer cell line. Using the Smad-specific binding sites identified from these experiments, we performed *in-silico* characterization of the structural features that dictate R-Smad specific binding, and concluded that local sequences encode significant amounts of information. Thereafter, we used deep learning methods to classify a pool of R-Smad-bound sequences into Smad2- or Smad3-bound. Finally, we took the CNN-LSTM hybrid model and used it to disambiguate the roles of Smad2 and Smad3 in early and late response to TGF *β*-1 in a separate breast cell line, MCF10A-MII.

Our *in-silico* structural predictions of Smad2 and Smad3 binding sites suggest that regions flanking Smad2 binding sites have wider minor groves as compared to Smad3 binding sites. This difference in minor grove in turn also correlates with a larger electrostatic potential in Smad2-bound sites. The structural differences can be attributed to differences in sequences. In turn, the difference in sequence can be traced to the different transcriptional co-regulating partners of R-Smad. As the structural properties are encoded by the sequence, we used the sequences to develop a neural network model to disambiguate Smad2 and Smad3 binding sites. Consistent with earlier studies, our data suggests that a CNN-LSTM hybrid model outperforms a CNN-only model in such classification tasks. Finally, we applied our model to disambiguate the roles of Smad2 and Smad3 in breast cancer disease progression from a publicly available dataset. Our functional enrichment analysis suggests differential roles of Smad2 and Smad3 in both early and late TGF *β* response, with a more pronounced role of Smad3 in sculpting the early response while both Smads regulate different processes involved in the epithelial-mesenchymal transition program in the late TGF *β* response.

While our results suggests the feasibility of using machine learning to disambiguate Smad2 and Smad3 binding sites, there are several limitations of the present model that represent potential avenues for improvement. First, our current model treats Smad2 and Smad3 binding as at distinct sites; future work to develop a multi-class model can be undertaken to identify sites to which both Smad2 and Smad3 can bind. A second limitation of our model is the lower generalizability observed in the hESC dataset. This is due to the lack of training data from other cell types, which leads to the inability of our model to learn more generalizable features of Smad2/Smad3 binding sites. More experimental data from Smad2/Smad3 specific ChIP in other cell types would be required in order for a more generalizable model to be developed.

## Methods and materials

### Molecular cloning

The BAC-SMAD2 and BAC-SMAD3 recombinant plasmids used in this study were provided by the Genome Engineering Core Facility of the Institute for Genomics and Systems Biology at the University of Chicago. A BAC containing the gene and endogenous *cis* control elements was tagged by recombineering to yield the Localization and Affinity Purification(LAP) tag at the C-terminus [18]. Smad2 was tagged in CH17-5E15BAC (BAC-SMAD2) while Smad3 was tagged in CH17-187G10BAC (BAC-SMAD3). Following expansion, the plasmids were extracted using the Maxi/BAC’ protocol with the Nucleobond AX 100 kit (Macherey-Nagel, Hoerdy, France).

### Cell culture

For the generation of cells stably expressing LAP-tagged Smad2/Smad3 (referred to as BAC-SMAD cells), MDA-MB-231 cells (ATCC HTB-26) were transfected with BAC-SMAD plasmid via Lipofectamine 2000. Selection of transfects was performed with Geneticin. Three weeks after antibiotic selection, the cells were GFP-selected using Moflo XDP Cell Sorter (Beckman Coulter) that was incorporated into a Class II BSC and equipped with the standard Ar and Kr gas lasers and a 488 nm 200 mW blue laser to obtain a highly purified BAC-SMAD population. The transfected cells were maintained in DMEM, 4500mg/L glucose supplemented with 10% (v/v) Fetal Bovine Serum, 100U/mL penicillin/ streptomycin and 800 *μ*g/mL Geneticin (Gibco) in a humidified 5% CO2 incubator at 37^∘^C.

### Western blot

Treatment of cells was performed with 10ng/mL of TGF- *β*1 (Sigma #T7039). Cells were lysed in RIPA Buffer containing protease inhibitors and phosphatase inhibitors and quantification was performed using Quick Start™ Bradford Protein Assay. The protein lysate were denatured and fractionated with NuPAGE Novex 4-12% Bis-Tris SDS-PAGE in 1X MES buffer. The resolved proteins were wet-transferred onto nitrocellulose membrane and blocked for one hour. The membrane was incubated overnight at 4^∘^C with primary antibodies. Antibodies used: Smad2 antibody abcam #ab71109, Smad3 antibody abcam #ab28379, Smad4 abcam #ab3219, GFP antibody abcam #ab290 and GAPDH Ambion #am4300. (Additional file 1) Visualization was performed with Amersham ECL Select Detecting Reagent with FluorChem R Imager (ProteinSimple, CA, USA).

### ChIP-sequencing

Chromatin Immunoprecipitation (ChIP) was performed with the EZ-Magna ChIP™ A Chromatin Immunoprecipitation Kit (Millipore, Billerica, MA, USA) with anti-GFP (abcam). ChIP DNA was purified with Qiagen PCR purification kit and quantification was performed using the Qubit$\circledR $ 3.0 Fluorometer with Qubit$\circledR $ dsDNA HS Assay Kit. DNA libraries were generated using the TruSeq ChIP sample Prep kit (Illumina) followed by deep sequencing with the Illumina’s HiSeq 2500 system with at least 100M (million) raw reads for a ≥ 40M clean single-end reads with a minimum requirement of target non-redundancy fraction (NRF) of ≥ 0.8 for 10M reads uniquely mapped read. Sequencing was performed at the Beijing Genome Institute (BGI).

### Bioinformatics analysis of ChIP-seq data

Sequencing reads were aligned to the hg38 genome using Bowtie2 [47]. Following alignment, peak calling was performed using MACS2, with reads extended to 200 base pairs to recover the original binding sites [48]. Downstream annotations and analysis was performed in R. Peaks identified by ChIP-seq data in TGF *β* treated MCF10A-MII was downloaded from GEO (accession number: GSE83788) [42]. Likewise, peaks from TGF *β* treated human embryonic stem cells were downloaded from GEO [41] (accession number: GSE29422) and peak coordinates were converted to hg38 using the liftover tool. Peak annotation and feature encoding was performed in a similar manner to our in-house dataset (described below). Gene ontology (GO) enrichment analysis was performed using ReactomePA [49] and default settings. GO terms with a *p*-value of less than 0.05 were considered to be enriched.

### Architecture of neural networks

**Fig. 4:**
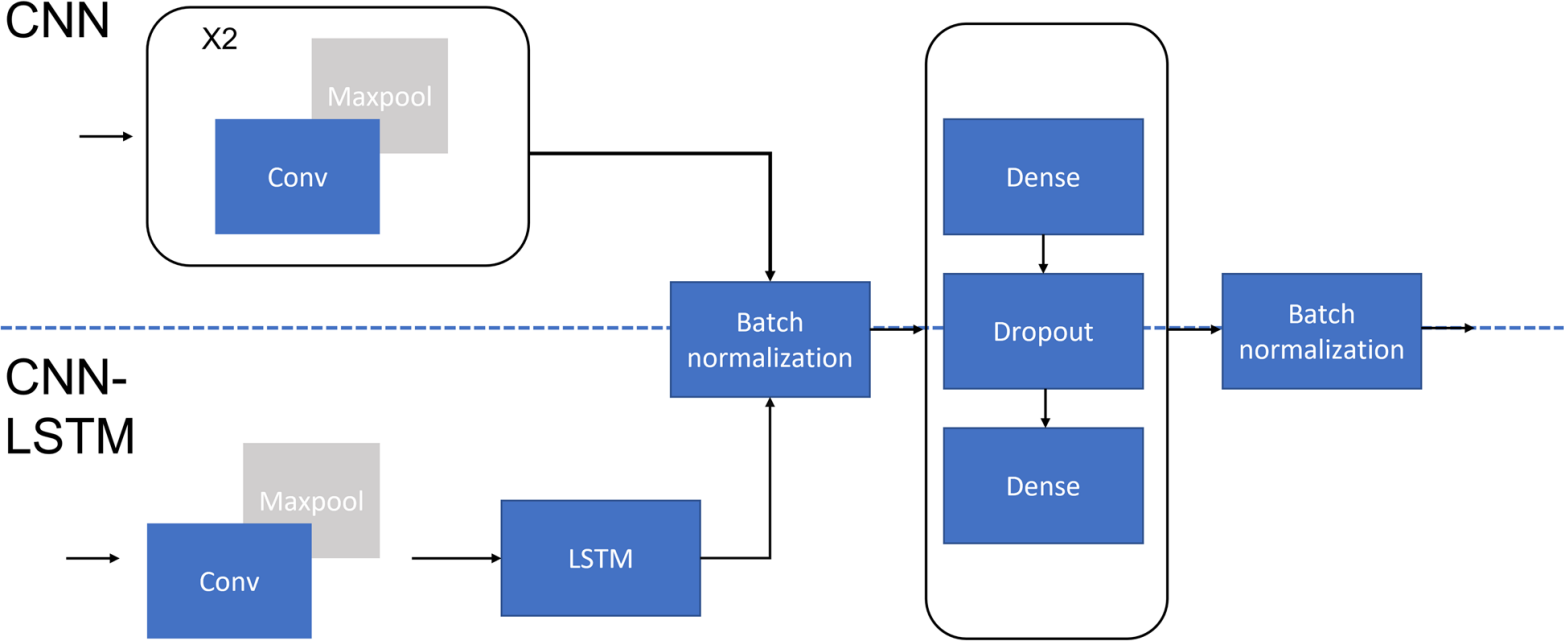
Architectures of neural networks used in this study. The CNN is made of two convolution stacks (convolution layer + maxpooling). A filter size of five is used in the first convolution stack to serve as a motif detector. Thereafter, we used a larger filter size (32) in the next convolutional layer to capture larger patterns in the sequence. Following the convolution stacks, the features are flattened and batch normalized before passing through two dense layers using the ReLu activation function which are connected by a drop out layer. Finally, the output from the dense layer is passed to an output layer with a sigmoid activation to produce a final prediction value. Similar to the CNN model, we first used a convolution layer with a filter size of five to serve as a local motif detector for our CNN-LSTM model. After maxpooling, the output matrix is passed to an LSTM with 32 cells. Thereafter, the output from the LSTM is batch normalized and passed through two fully connected layers with the same configuration as our CNN model

Various neural network architectures have been proposed for the task of TFBS prediction, with CNN and RNN as the two dominant architectures. Various forms of RNNs have been proposed, with long-short term memory (LSTM) and gated recurrent units (GRU) as two dominant types of RNNs used in TFBS prediction. Two models were trained - a vanilla CNN model comprising only of convolutional layers connected to two fully connected layers, and a CNN-LSTM hybrid comprising a convolutional input layer connected to a long-short term memory (LSTM) layer before being connected to two fully connected layers. Figure 4 shows the configurations of our models. A dropout layer with a dropout ratio of 0.2 was added between the two dense layers to prevent overfitting.

For the two fully connected layers prior to the output layer, the number of neurons chosen was determined based on the work by Huang et al. [50], which specified the minimum number of neurons required to capture all the samples within the dataset. This allows us to choose the smallest possible number of neurons in the dense layers while not losing valuable information for model training. We used the ReLu activation function for each layer in the fully connected layers prior to passing the values to the output layer using the sigmoid function to obtain a final predicted value.

### Model training and evaluation

Smad2/3 bound promoters (defined to be within 3kb of transcription start sites) were first resized to 200 base pairs. Thereafter, the *N* sequences were one-hot encoded to produce a *N*×200×5 matrix which was then used as input for training and prediction. In our one-hot encoded matrix, each promoter is encoded in one of *N* rows. Each base is encoded by 5 slices corresponding to either A,T,C,G or N. Neural network training was performed in Keras using the Tensorflow framework. Training was performed with 75% of the dataset, with the other 25% reserved for model testing. We used a cosine annealing training schedule with restarts [51], where the learning rate was gradually decreased in each epoch according to the formula

$$a(t) = \frac{a_{0}}{2}\left[cos\left(\frac{\pi|(t-1,T/M)|}{T/M}\right)+1\right]$$

where *a*(*t*) refers to the learning rate at epoch *t*, *a*_0_ refers to the maximum learning rate, and *T* and *M* represents the total number of epochs and number of training cycles respectively.

We combined the cosine annealing training schedule with snapshot ensemble learning [52], where the outputs from ten different models are averaged to produce a final predicted value. The learning rate was reset to the maximum learn rate at the start of each model. The area under the precision recall curve (AUPR) was used as the metric of model performance. As our dataset was highly imbalanced with 75% of the sites being Smad3-bound, we used a cut-off probability of 0.75 for classifying peaks as being Smad2 or Smad3 bound.

## Supplementary Information

**Additional file 1** Western blots of the Smad and LAP-Smad constructs as described in the text, along with original gel images. Images from the Fluorchem R imaging system are tuned for band exposure, and the edges of gels may not be visibile.

**Additional file 2** LAP-tag ChIP-seq peaks compared to peaks called using commercially available SMAD3-specific antibodies in untreated (left) and treated (right) samples. Very low *p* value peaks are highly concordant.

## Data Availability

Source code and data files used for the neural network can be found at https://bitbucket.org/jeremy_ng/incob-2021-ng_et_al. ChIP seq data is available at GEO under accession GSE190237.

## Abbreviations

AUPR: Area under precision-recall curve
ChIP-seq: Chromatin immunoprecipitation sequencing
CNN: Convolutional neural network
GFP: Green fluorescence protein
GO: Gene ontology
LAP: Localization and affinity purification
LSTM: Long-short term memory
RNN: Recurrent neural network
R-Smad: Receptor regulated Smads
SBE: Smad-binding element
TFBS: Transcription factor binding site
TGF- *β*: Transforming Growth Factor Beta
