# Supplementary material for: Deep learning for de-convolution of Smad2 versus Smad3 binding sites
Source: BMC Genomics. 2022 Jul 20;23(Suppl 1):525. doi: 10.1186/s12864-022-08565-x (PMC9297549; doi:10.1186/s12864-022-08565-x)

Additional file 1 Western blots as described in the text

Ng et al

Western blots of the Smad and LAP-Smad constructs as described in the text.

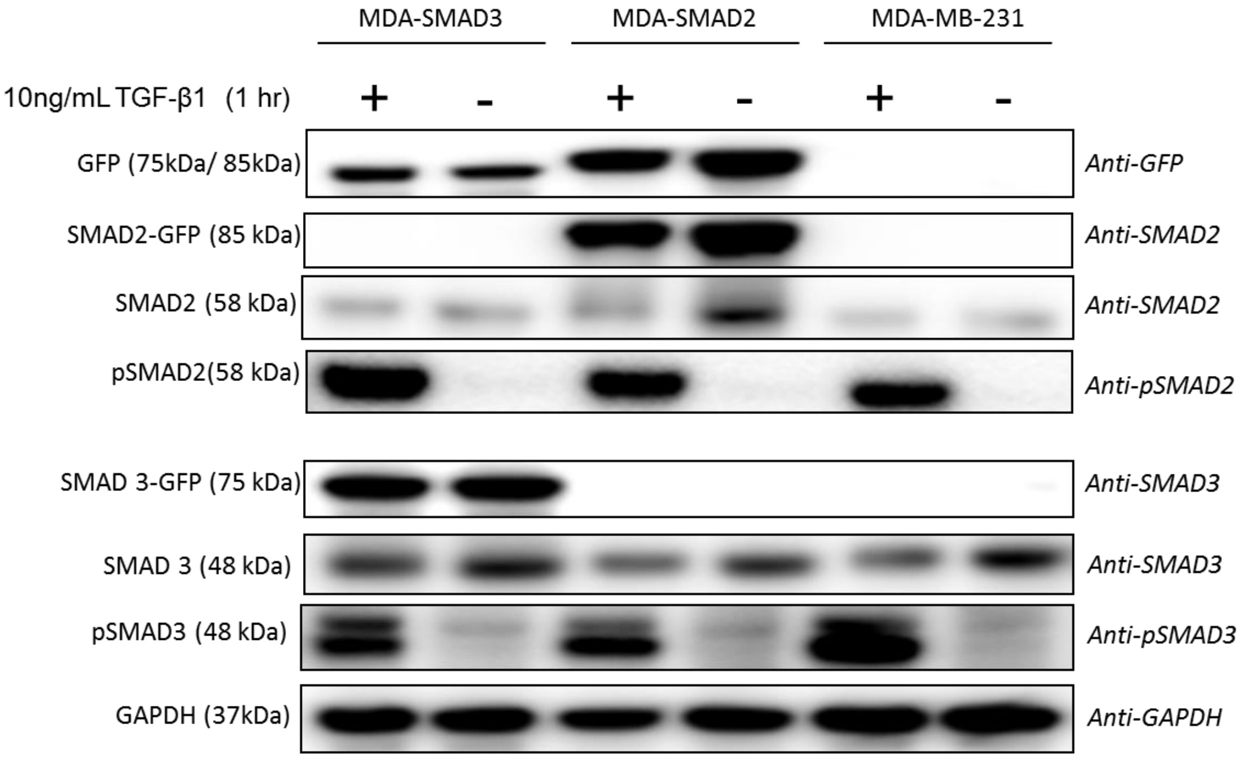

Smad3 original gel image

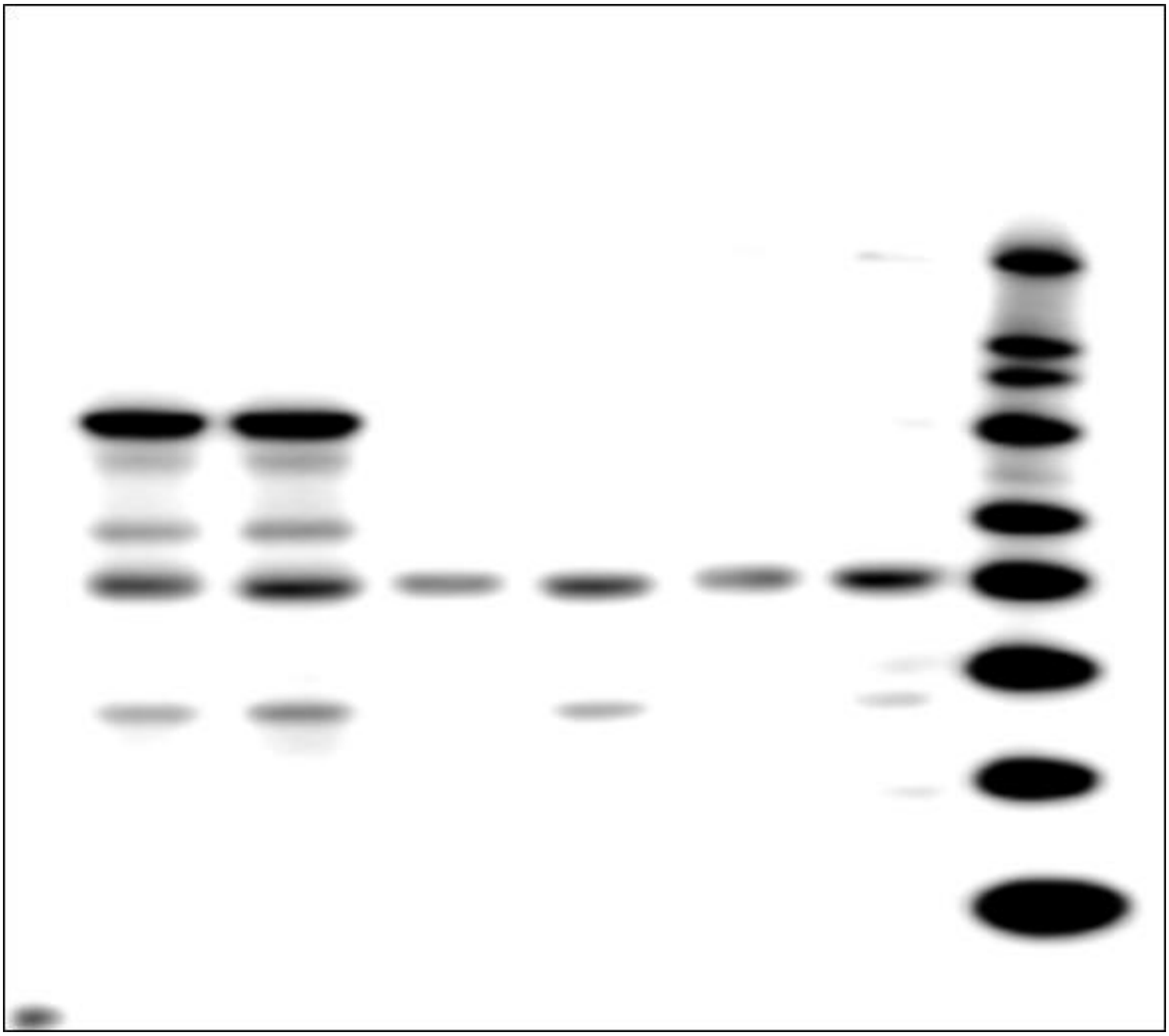

Smad2 original gel image

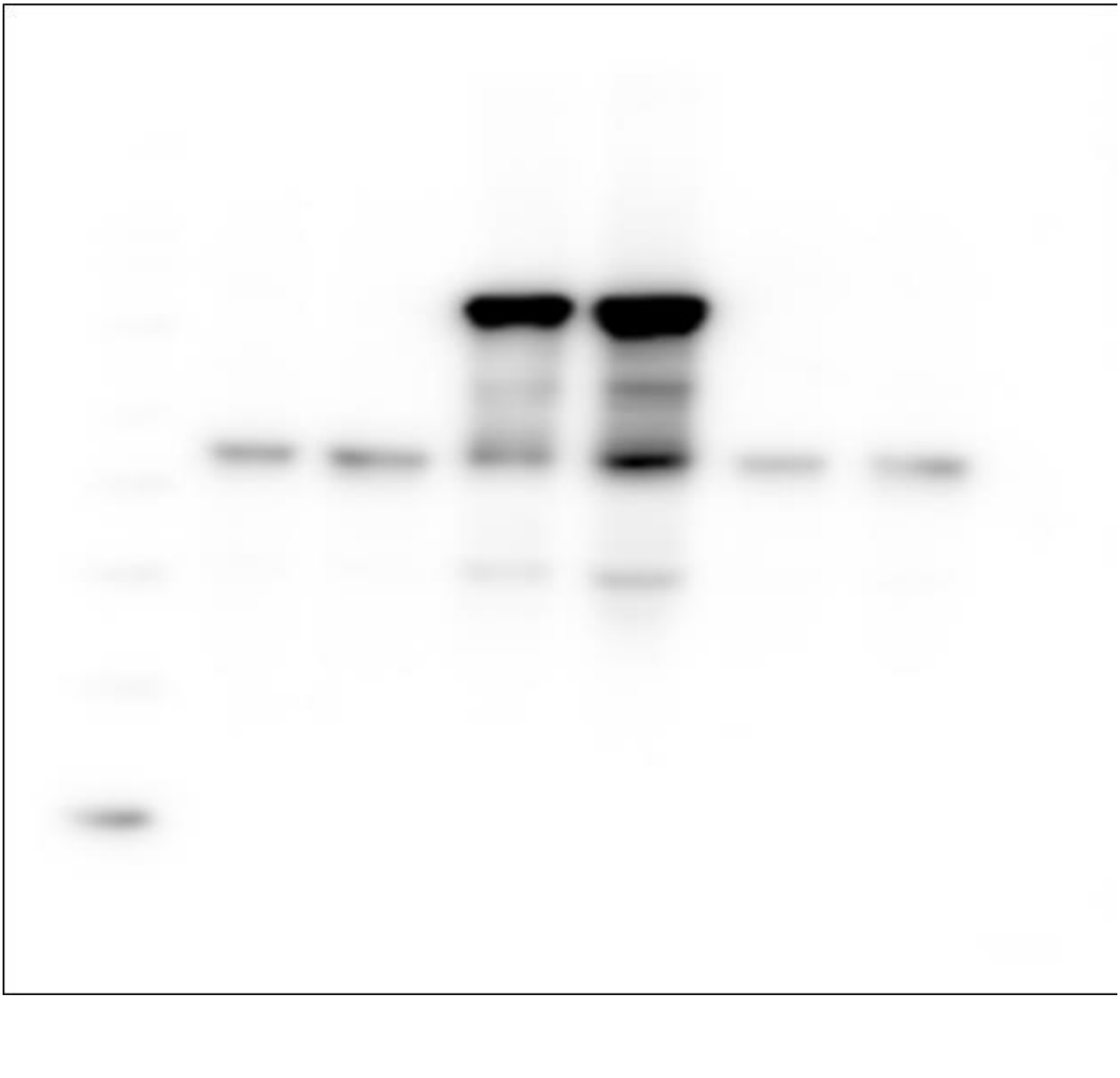

GAPDH original gel image

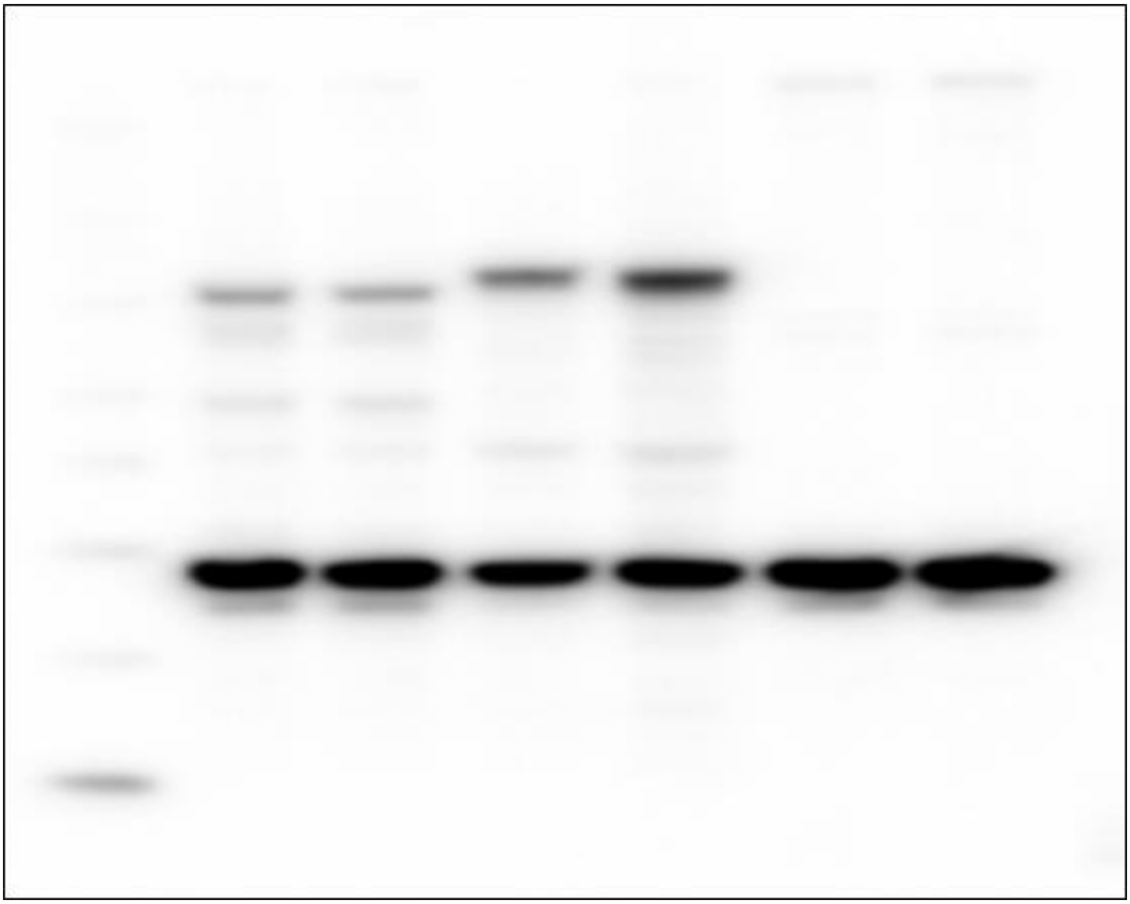

pSmad3 original gel image

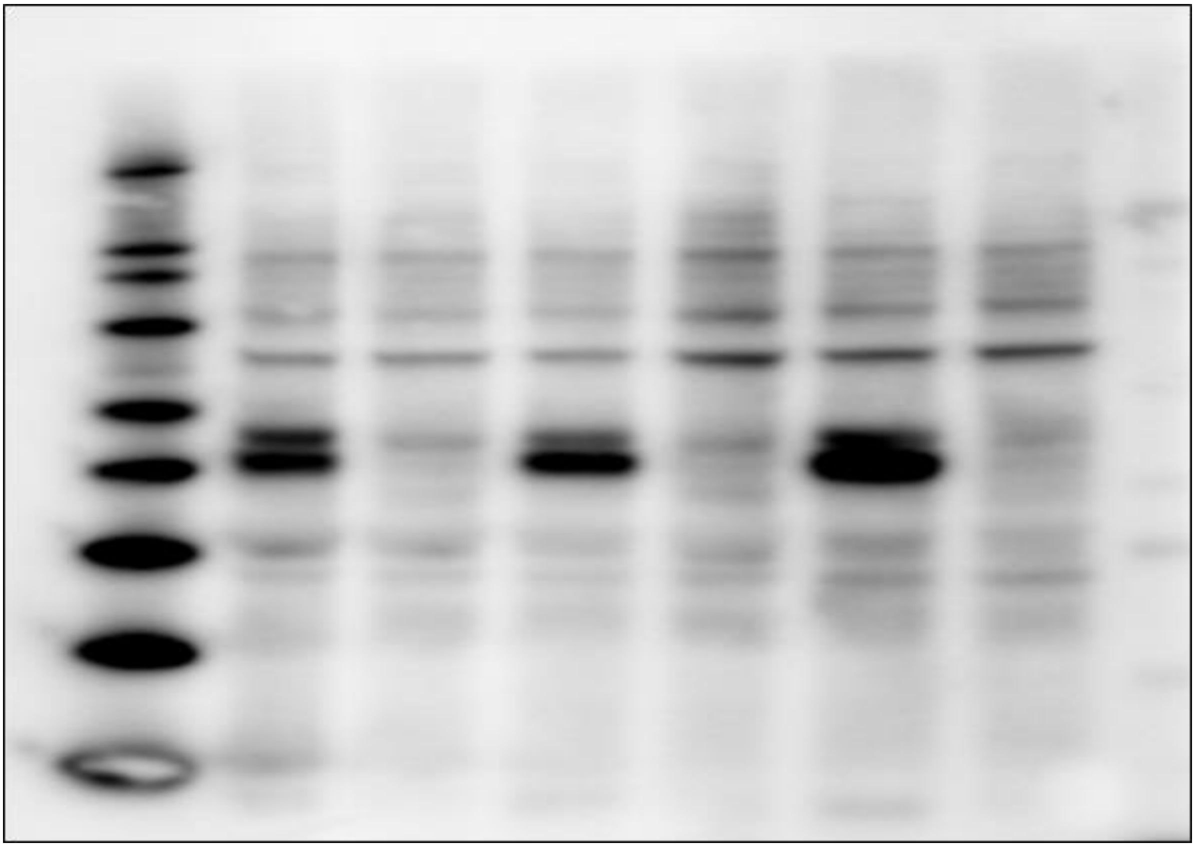

pSmad2 original gel image

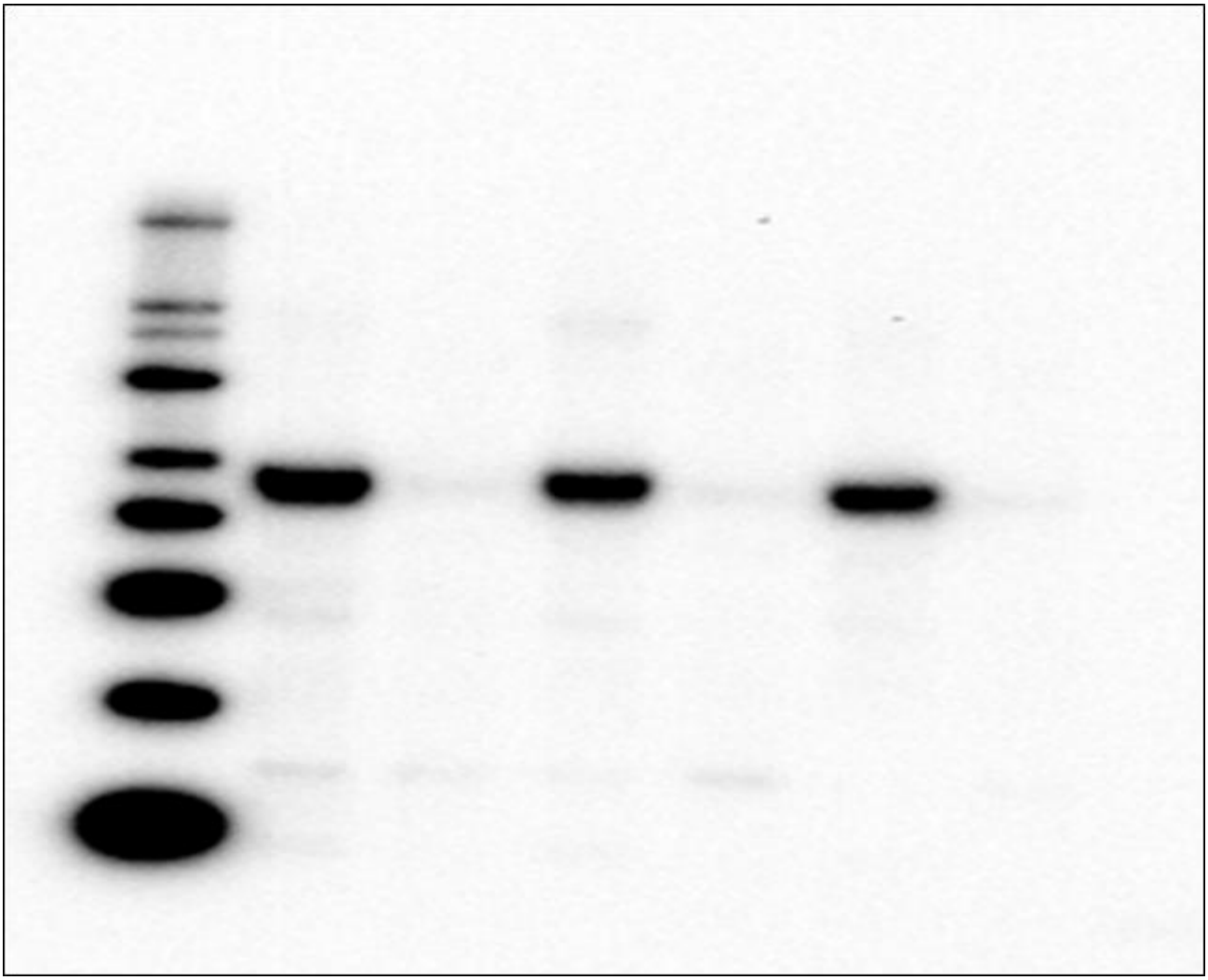

GFP original gel image

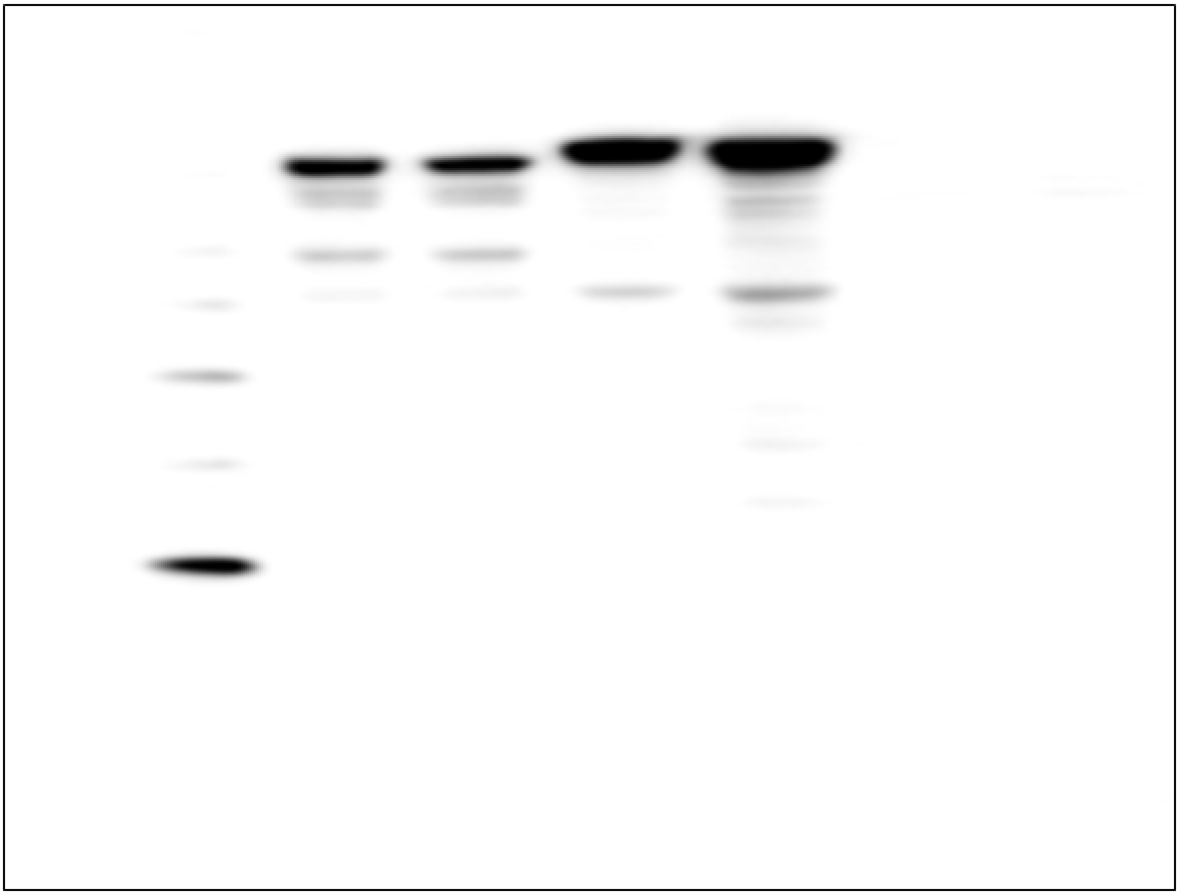

Supplement: Supplementary file 1 — Additional file 1 Western blots of the Smad and LAP-Smad constructs as described in the text, along with original gel images. Images from the Fluorchem R imaging system are tuned for band exposure, and the edges of gels may not be visibile. [file 12864_2022_8565_MOESM1_ESM.pdf]
