# Supplementary figures and images for: Deep learning for de-convolution of Smad2 versus Smad3 binding sites
Source: BMC Genomics. 2022 Jul 20;23(Suppl 1):525. doi: 10.1186/s12864-022-08565-x (PMC9297549; doi:10.1186/s12864-022-08565-x)

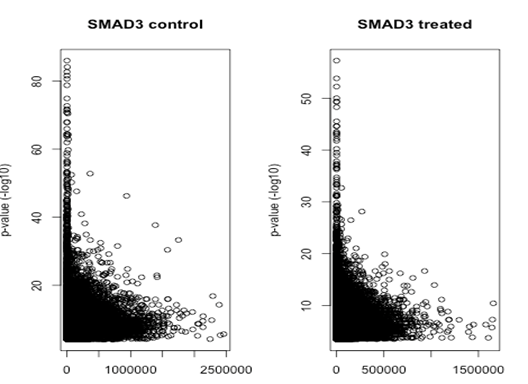

Supplement: Supplementary file 2 — Additional file 2 LAP-tag ChIP-seq peaks compared to peaks called using commercially available SMAD3-specific antibodies in untreated (left) and treated (right) samples. Very low p value peaks are highly concordant. [file 12864_2022_8565_MOESM2_ESM.png]
